# Weight Loss Following Use of a Smartphone Food Photo Feature: Retrospective Cohort Study

**DOI:** 10.2196/11917

**Published:** 2019-05-29

**Authors:** Daniela Ben Neriah, Allan Geliebter

**Affiliations:** 1 Institute of Human Nutrition Columbia University New York, NY United States; 2 Touro College of Osteopathic Medicine New York, NY United States; 3 Department of Psychology Touro College and University System New York, NY United States; 4 Department of Psychiatry Mount Sinai St. Luke’s Icahn School of Medicine at Mount Sinai New York, NY United States

**Keywords:** food intake, digital photography, app tracking, dietary assessment, free-living

## Abstract

**Background:**

Tracking of dietary intake is key to enhancing weight loss. Mobile apps may be useful for tracking food intake and can provide feedback about calories and nutritional value. Recent technological developments have enabled image recognition to identify foods and track food intake.

**Objective:**

We aimed to determine the effectiveness of using photography as a feature of a smartphone weight loss app to track food intake in adults who were overweight or obese.

**Methods:**

We analyzed data from individuals (age, 18-65 years; body mass index≥25 kg/m^2^; ≥4 days of logged food intake; and ≥2 weigh-ins) who used a mobile-based weight loss app. In a retrospective study, we compared those who used the photo feature (n=9871) and those who did not use the feature (n=113,916). Linear regression analyses were used to assess use of the photo feature in relation to percent weight loss.

**Results:**

Weight loss was greater in the group using the photo feature (Δ=0.14%; 95% CI 0.06-0.22; *P*<.001). The photo feature group used the weight loss app for a longer duration (+3.5 days; 95% CI 2.61-4.37; *P*<.001) and logged their food intake on more days (+6.1 days; 95% CI 5.40-6.77; *P*<.001) than the nonusers. Mediation analysis showed that the weight loss effect was absent when controlling for either duration or number of logged days in the program.

**Conclusions:**

This study was the first to examine the effect of a food photo feature to track food intake on weight loss in a free-living setting. Use of photo recognition was associated with greater weight loss, which was mediated by the duration of app use and number of logged days in the program.

## Introduction

More than two-thirds of adults are considered to be overweight or obese in the United States [1]. Individuals with obesity are at an increased risk of developing cardiovascular disease, type 2 diabetes, and hypertension and are at a greater risk of mortality [2,3]. Weight loss can reduce the severity of these comorbidities or help prevent them [4,5]. Lifestyle modifications including calorie restriction can be effective in reducing body weight in the short term, although such approaches are less successful in the long term: Only about 20% of overweight individuals were successful at long-term weight loss, defined as losing at least 10% of the initial body weight and maintaining the weight for at least 1 year [6]. The current obesity epidemic has generated a large market for weight loss programs. Commercial weight loss programs offer various aids, including mobile apps that allow tracking of food intake.

The majority of the US adult population (77%) reported owning a smartphone device in 2018 [7]. Tracking of food intake by using a mobile app can provide instant feedback about the calories and nutrients of the meal. Dietary self-monitoring is a key component in weight loss programs [8], and frequency of self-monitoring is strongly correlated with weight loss [9,10]. Some studies have reported a modest, although significantly greater, weight loss associated with the use of mobile apps compared to more traditional methods such as pen and paper [11-15], whereas others have found no difference [16-18]. Meta-analyses and systematic reviews of the recent literature also report mixed results, partly due to variation in the inclusion criteria [19,20]. In one meta-analysis, Flores-Mateo et al [19] reported significantly greater weight loss among people using mobile apps as compared to those using other methods, whereas Semper et al [20] reported no significant difference in their meta-analysis.

Self-monitoring requires time and effort, and many find tracking of dietary intake tedious, which contributes to attrition [21]. Obesity researchers and weight loss companies have attempted to improve the ease of tracking by offering new features. One such feature employs photography of food items to monitor dietary intake both in the clinical setting and everyday life [22-25]. Since the technology has only been developed recently, the literature on this subject is limited. However, a few studies have examined the effect of using photography to record food intake. A method developed by Martin et al [26], called Remote Food Photography Method (RFPM), uses semiautomatic computer analysis performed by researchers to obtain nutritional value from food images submitted by users. However, due to technological limitations, this process needs to be overseen by trained professionals [27,28]. Recent studies using RFPM conducted in pregnant women and preschoolers with obesity found that this method was not accurate in estimating energy intake compared to doubly labeled water [29,30]. Doumit et al conducted a cross-over study in college students, mostly of normal weight, to examine the effects of recording food intake from memory or with the aid of cell phone photos on energy intake and food choices [31]. They reported a nonsignificant trend for lower energy intake in the group using cell phone photos, suggesting increased awareness of food choice and portion size. The study did not assess weight change and was of a short duration, with 3 days for each assessment period. A retrospective cohort study examined factors related to the use of food photography with an app that promotes healthy eating [32]. Active users (with 10 photos or more) had, on an average, used the app for longer and had higher healthy ratings per photo than nonactive users (1 photo) and semiactive users (2-9 photos). However, this study did not monitor weight change.

Recently, a new tool, Snap It™, became available that allows participants to take photos of their food, and through image recognition technology attempts to match the food item to a large food photo database [33]. We investigated the effect of using a food image recognition feature as part of a mobile app for tracking food intake on weight loss. We hypothesized that the photo feature users would lose more weight than nonusers.

## Methods

### Mobile App

Lose It! is a free weight loss mobile app launched in 2008 by FitNow (Boston, MA), which allows users to record their daily food intake. Users enter their self-reported weight, height, gender, and age when they sign up. They choose their goal weight and the rate at which they would like to lose weight (0-2 lbs per week). The app allows users to record food intake and exercise, calculates calories consumed, and estimates calories expended. All users have a calculated calorie budget for the day and can see whether their intake was within their budget. Users can also choose to pay US $39.99 per year for the premium features, which offer meal and exercise planning, macronutrient tracking, and recipes. In November 2016, a new free feature was added to the app that utilizes a food image tool called Snap It, which recognizes food items and requests the user to confirm their meal items from a list of potential matching foods and to add estimated portion sizes. The feature became available to all users of the app, but only a fraction have used it. We investigated weight loss outcomes among those who used the app versus those who did not. The data were de-identified before they were provided to us by the company FitNow, which had no role in the development of the protocol, the interpretation of the data, or the preparation of the manuscript.

### Photo Feature

**Figure 1:**
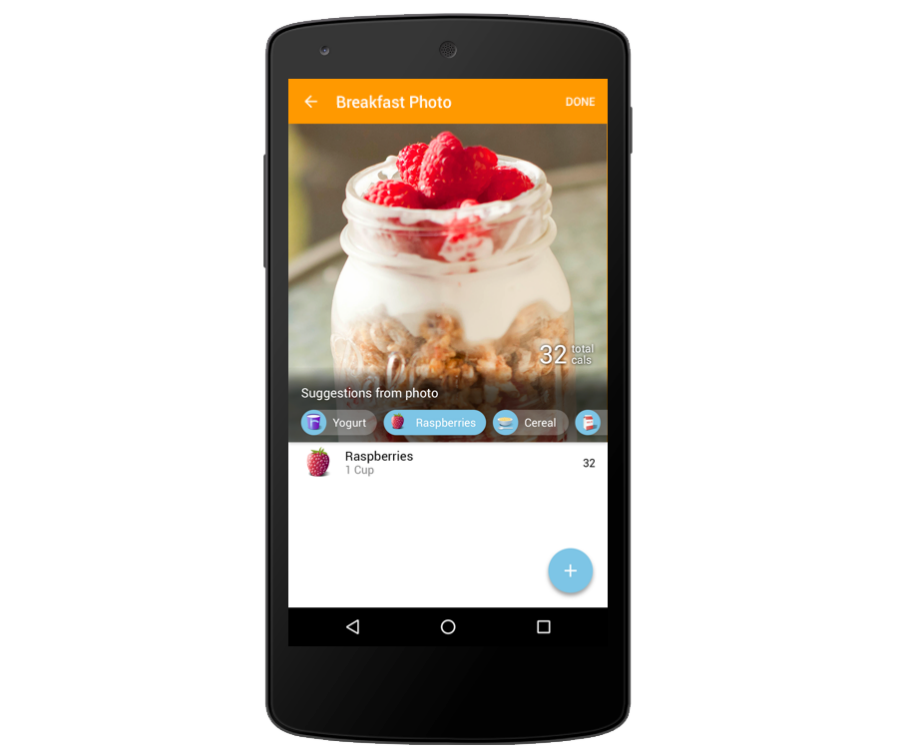
App photo feature. Users can take a snapshot of their food items before eating and select correct matches among a list of suggested items [33]. The calories and nutrients for the items are then displayed.

The Snap It photo feature is shown in Figure 1 and is available to all app users at no additional cost. The user takes a photo of food items and then obtains a list of food items that the software recognizes as potential matches. The user subsequently chooses the correct match and specifies the quantity consumed from the list. Thereafter, the calories and macronutrients are calculated and displayed.

### Study Sample

**Figure 2:**
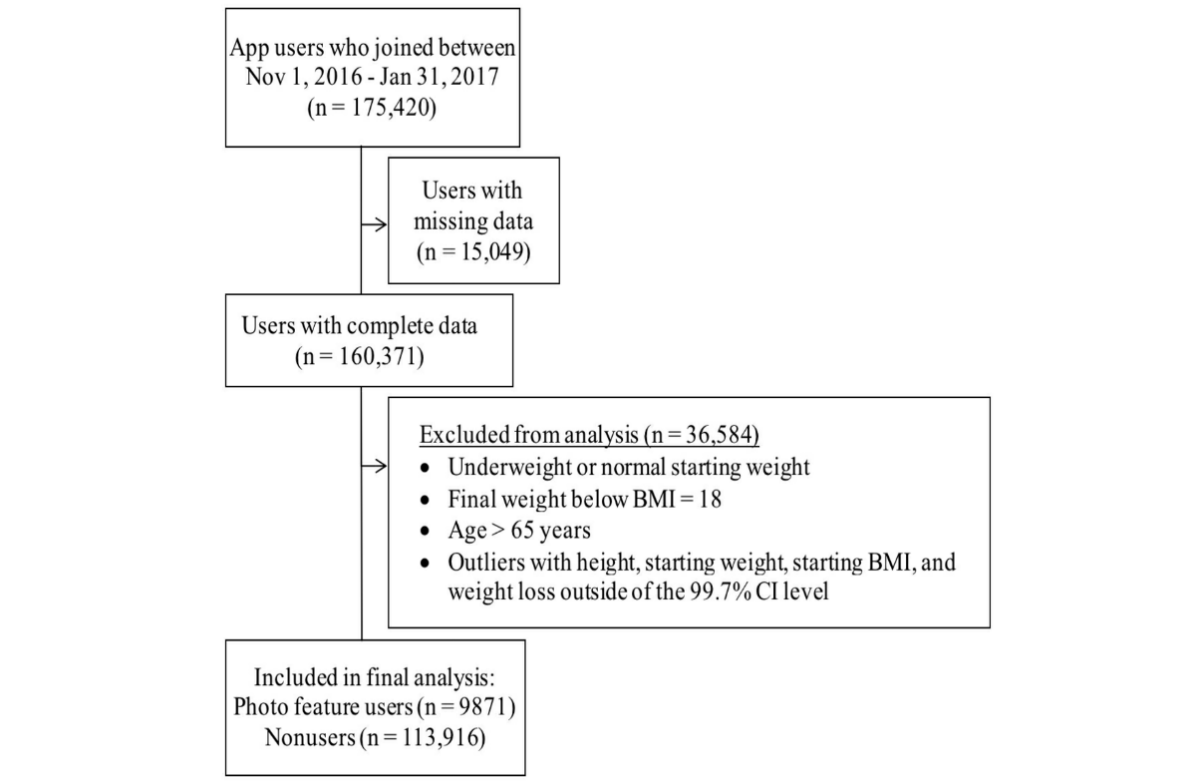
Flow chart of Lose It! app users in the study. BMI: body mass index.

We received the dataset from FitNow, Inc, which included 175,402 users who joined between November 1, 2016, and January 31, 2017. Data collection for this sample ended in April 2017 and included individuals who had logged food intake in the app for a minimum of 4 days; had at least two reported weigh-ins; had a date of birth between January 11, 1936, and April 21, 1999; and reported >3 feet in height and a body weight between 70 and 750 pounds. The study was approved by the Touro College and University System Institutional Review Board (HSIRB# 1746E). We analyzed individuals who were overweight or obese, defined by a starting body mass index (BMI)≥25 kg/m^2^ [34], with a final BMI≥18 kg/m^2^ and age of 18-65 years. To exclude users with unrealistic data, we removed outliers outside the 99.7% CIs for height, starting weight, starting BMI, and %weight change. Only users who used an iPhone or an Android phone were included, thus excluding Web users, as the photo feature is not available on the LoseIt! website. Usage duration was calculated based on the number of days between the first and last reported weigh-ins, and the number of “logged days” was based on the number of days that intake was entered in the app. The groups were defined as photo feature users if they used the photo feature to log food intake for at least 1 or more days, and as nonusers if they did not use the photo feature at all. Only 7.8% (13,663/175,402) had used the photo feature, likely because the feature was new. We then excluded users who did not meet the inclusion-exclusion criteria or had missing or outlier data. We analyzed 123,787 users overall (Figure 2), grouped by those who used the photo feature (n=9871) and those who did not use the feature (n=113,916). Among the photo feature users, we examined weight change in relation to the number of days the photo feature was used to log food intake.

### Statistical Analysis

We applied regression analysis using RStudio (version 1.0.14, RStudio Team, Boston, MA) to compare the %weight loss for the photo users and nonusers as well as duration in the program and number of logged days. We included the covariates, starting BMI, gender, age, premium status, and type of user operating system (Android or iPhone). We then reanalyzed the %weight loss data and controlled for duration and number of logged days. Chi-squared tests and *t* tests were used for baseline characteristics and comparisons between groups. Results were considered significant if the *P* value was ≤.05. The SD or 95% CI was used to represent the variance around the mean.

## Results

### Study Sample

Table 1 shows the composition of the study sample and the baseline characteristics of the participants, including age, starting weight, starting BMI, type of operating system (Android or iPhone), premium subscription status, and goal weight. Nonphoto feature users were younger, had a lower BMI and a lower body weight, used iPhones more often (vs Android), and paid for the premium version of the LoseIt! app less often than photo feature users.

**Table 1:** Baseline characteristics and comparisons between groups*.*

Characteristic	Nonusers (n=113,916)	Users (n=9871)	*P* value
Age (years), mean (SD)	36.1 (12.1)	36.5 (11.9)	<.001
Starting weight (kg), mean (SD)	93.6 (20.5)	94.0 (20.6)	.051
Starting body mass index (kg/m^2^), mean (SD)	32.7 (6.2)	33.1 (6.3)	<.001
Female, n (%)	83,150 (72.99)	7153 (72.46)	.26
Android, n (%)	28,041 (24.62)	3438 (34.83)	<.001
iPhone, n (%)	85,875 (75.38)	6433 (65.17)	<.001
Premium, n (%)	13,283 (11.66)	1834 (18.58)	<.001
Goal weight loss (kg), mean (SD)	8.7 (6.2)	8.9 (6.3)	.001

### Weight Loss

Reported body weight decreased over time across the two groups, with greater weight loss in the photo feature group. The photo feature users lost 0.14% more (mean 3.42%; 95% CI 3.27-3.58) than the nonusers (mean 3.28%; 95% CI 3.24-3.32; *P*<.001), which translated to a 0.15 kg difference (photo feature users: mean weight loss=3.29 kg, 95% CI 3.14-3.45; nonusers: mean weight loss=3.14 kg, 95% CI 3.10-3.19). The difference in the %weight loss remained significant after adjusting for starting BMI, age, gender, user operating system, and premium status (*P*=.002), which were all significantly associated with the %weight loss. When adjusted for duration, photo feature use was not significantly associated with %weight loss (*P*=.40), and when adjusted for the number of logged days, the weight loss effect was reversed, where photo feature use was associated with an increase in body weight (+0.18 kg; *P*<.001). Duration and number of logged days were both significantly associated with %weight loss (*r*=0.33, *P*<.001 and *r*=0.46, *P*<.001, respectively). Collinearity between duration and number of logged days was not significant (variation inflation factor=1.37).

Within group analysis of photo feature users only showed that the number of days the photo feature was used was significantly associated with the %weight loss (*P*<.001). The difference was associated with 0.04% (95% CI 0.02-0.06) weight loss for every additional day of using the photo feature.

### Premium Version

Of the 123,787 users analyzed, 15,117 (12.2%) were premium users and 1834 (12.1%) of the premium users used the photo feature. Premium users had a higher starting weight (96.4 kg; 95% CI 95.6-97.2) than nonpremium users (93.2 kg; 95% CI 93.0-93.4; *P*<.001). Premium users also lost more weight (mean 3.77 kg; 95% CI 3.60-3.93) than nonpremium users (mean 3.23 kg; 95% CI 3.19-3.27; *P*<.001). The difference remained significant even after adjusting for starting BMI, age, gender, and user operating system.

### Duration

The photo feature group used the app for 3.5 days more than nonusers (photo feature users: mean 59.0 days, 95% CI 57.3-60.7; nonusers: mean 55.5 days, 95% CI 55.1-56.0; *P*<.001). The difference remained significant (3.2 days; *P*<.001) after adjusting for starting BMI, age, gender, user operating system, and premium status (*P*<.001).

### Number of Logged Days

Photo users logged 6.1 more days than nonusers (users: mean 43.1 days, 95% CI 41.7-44.5; nonusers: mean 37.0 days, 95% CI 36.6-37.4). The difference remained significant (5.4 days, *P*<.001) after adjusting for starting BMI, age, gender, user operating system, and premium status.

### Mediation Analysis

**Figure 3:**
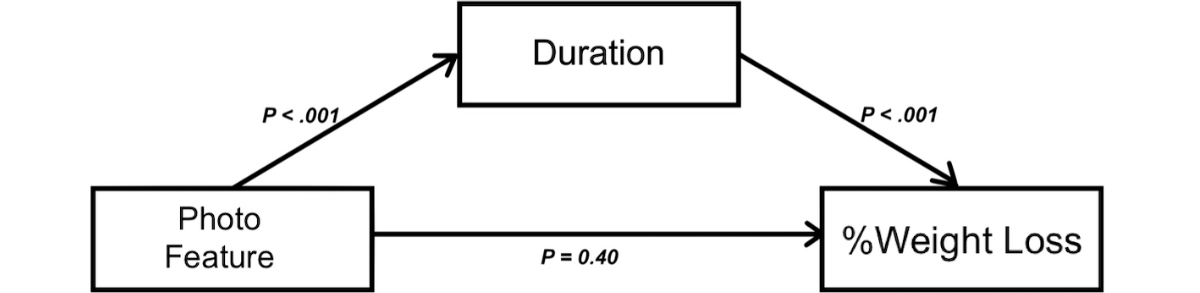
Duration as a mediator. The photo feature use was significantly associated (P<.001) with weight loss before adding the potential mediator, duration, as a covariate. The photo feature use was also significantly associated with duration (P<.001), and duration was significantly associated with weight loss (P<.001). When adjusting for duration, the photo feature use was no longer significantly associated with weight loss (P=0.40), indicating that duration was a mediator.

**Figure 4:**
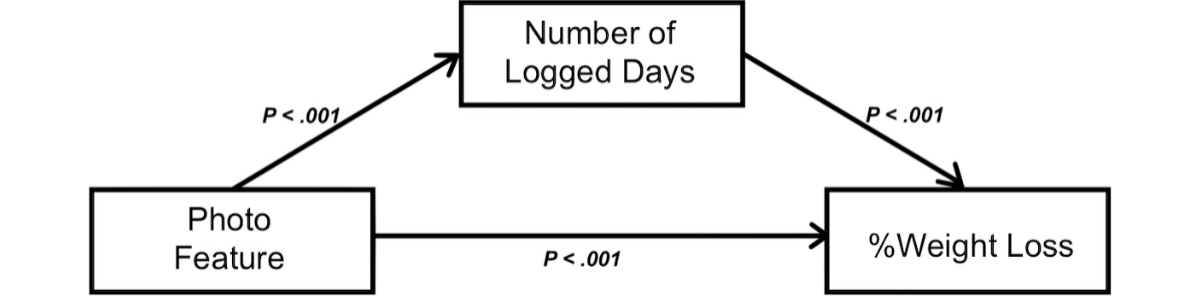
Number of logged days as a mediator. The photo feature use was significantly associated with weight loss (P<.001) before adding the potential mediator, number of logged days, as a covariate. The photo feature was also significantly associated with the number of logged days (P<.001), and the number of logged days was significantly associated with weight loss (P<.001). Direction of significance was reversed when adjusted for the number of logged days (P<.001), indicating that the number of logged days was a mediator.

As reported above, %weight loss was significantly associated with photo feature use (*P*<.001). Duration was also significantly associated with photo feature use (*P*<.001) and %weight loss (*P*<.001). When controlling for duration in the program, the effect of photo feature use on weight loss became nonsignificant (*P*=.40), indicating that duration was a mediator (Figure 3). The number of logged days was also significantly associated with photo feature use (*r*=0.05; *P*<.001) and %weight loss (*P*<.001). The effect on %weight loss was reversed (ie, photo feature use was associated with an increase in body weight) when the number of logged days was controlled for (+0.18 kg; *P*<.001), indicating mediation (Figure 4). Thus, both duration and number of logged days were significant mediators of the photo feature effect on %weight loss.

## Discussion

### Primary Outcomes

In this study, we analyzed data from 123,787 users of a weight loss app, which included an optional photo feature. Photo feature users lost significantly more weight than nonusers after adjusting for starting BMI, age, gender, user operating system, and premium status, which confirmed our hypothesis. Use of the photo feature might be less time consuming, more motivating, and more interactive than typing in food items.

Although statistically significant, the difference in weight loss between the groups was not clinically significant, indicating only a very small benefit of capturing food images by a phone camera. In addition, the photo feature users had more logged days and longer duration in the program, which may be due to enhanced motivation derived from taking photos, such as greater awareness of choice of food and portion size. Photographing food might also improve memory of the food eaten, which has been associated with reduced food intake [35]. On the other hand, individuals who were more motivated initially may have opted to use the photo feature.

The weight loss effect was not significant when adjusted for duration in the program and was reversed when adjusted for number of logged days. Mediation analysis showed that the weight loss differences were mediated by duration and number of logged days. Since entering the number of logged days as a covariate reversed the direction of significance (ie, photo feature was associated with weight gain when adjusted for number of logged days), the number of logged days appears to more strongly mediate the effect than duration in use of the photo feature and weight loss. As previously shown, increased self-monitoring, such as that by recording food intake, correlates with weight loss [36]. Thus, as the photo users remained in the program for a longer duration and logged more days, they spent more time recording their food intake.

An additional possible benefit to recording food intake by taking photos as compared to typing it into an app is that the photo is taken before the food is eaten, whereas typing in a food item is customarily done at end of the meal or end of the day, based on memory [37]. Thus, taking a photo prompts the user to acknowledge the nutritional content of the food item before consuming it, which may have a greater influence on their choice of food and portion size.

The frequency of photo feature use, reflected by the number of days of use of the photo feature, was significantly associated with weight loss, indicating that the more the photo feature was used, the greater the amount of weight lost. The photo feature users’ adherence, reflected in the number of logged days, was also higher than that of nonusers, suggesting that either the use of the photo feature motivated the users to continue with the program or that the users were more motivated to start with and used this feature as an additional tool. The latter theory is also supported by the increase in the proportion of photo feature users that subscribed to the premium features. As reported above, increased frequency of app use is correlated with increased weight loss and better maintenance [38]. Use of the photo feature was associated with increased weight loss, as hypothesized, possibly due to greater frequency of app use leading to a more successful outcome. This is the first study to examine the effect of a food image recognition app feature to track food intake on weight loss in a large cohort in a naturalistic, free-living setting.

### Limitations

There were several limitations, including limited demographic information, which did not include income and race. This study was also based on self-reported values of body weight and height. Additionally, the groups were self-selected and the numbers were uneven. In future studies, we would add a survey to gather demographic information and feedback on the ease of use and motivation to use the app with the photo feature. We observed a significant correlation between %weight loss and photo feature use, but this observational study does not allow determination of cause and effect. The next logical step would be a randomized controlled trial to help determine causal direction.

### Conclusions

We showed that use of a photo feature as part of a weight loss app was associated with greater weight loss, an effect mediated by increased duration and more logged days.

## Abbreviations

BMI: body mass index
RFPM: Remote Food Photography Method
